# Evidence of Placental Autophagy during Early Pregnancy after Transfer of *In Vitro* Produced (IVP) Sheep Embryos

**DOI:** 10.1371/journal.pone.0157594

**Published:** 2016-06-21

**Authors:** Paola Toschi, Marta Czernik, Federica Zacchini, Antonella Fidanza, Pasqualino Loi, Grażyna Ewa Ptak

**Affiliations:** 1 Faculty of Veterinary Medicine, University of Teramo, Piazza Aldo Moro 45, 64100, Teramo, Italy; 2 Institute of Genetics and Animal Breeding, Polish Academy of Sciences, Jastrzebiec ul. Postepu 36A, 05–552 Magdalenka, Poland; 3 National Research Institute of Animal Production, 1, Krakowska Street, 32–083 Balice n/Krakow, Poland; Faculty of Animal Sciences and Food Engineering, University of São Paulo, BRAZIL

## Abstract

Pregnancies obtained by Assisted Reproductive Technologies (ART) are associated with limited maternal nutrient uptake. Our previous studies shown that *in vitro* culture of sheep embryos is associated with vascularization defects in their placentae and consequent reduction of embryo growth. Autophagy is a pro-survival cellular mechanism triggered by nutrient insufficiency. Therefore, the goal of our present study was to determine if autophagy is involved in early placental development after transfer of *in vitro* produced (IVP) embryos. To do this, placentae obtained following transfer of IVP sheep embryos were compared with placentae obtained after natural mating (control—CTR). The placentae were collected on day 20 post-fertilization and post-mating, respectively, and were analyzed using molecular (qPCR), ultrastructural and histological/immunological approaches. Our results show drastically increased autophagy in IVP placentae: high levels of expression (p<0.05) of canonical markers of cellular autophagy and a high proportion of autophagic cells (35.08%; p<0.001) were observed. We conclude that high autophagic activity in IVP placentae can be a successful temporary counterbalance to the retarded vasculogenesis and the reduction of foetal growth observed in pregnancies after transfer of IVP embryos.

## Introduction

Since the first successful application of *in vitro* fertilization in 1978, the use of assisted reproductive technologies has contributed to the births of over 5 million babies worldwide and is expanding rapidly [1]. Although the majority of pregnancies after transfer of *in vitro*-produced (IVP) embryos result in normal, healthy outcomes, an increased risk for perinatal complications (e.g., preterm birth, smallness for gestational age), as compared to naturally conceived pregnancies, has been reported [2–4]. Many pregnancy-related complications observed late in gestation, such as pre-eclampsia and intrauterine growth restriction (IUGR), appear to have their origins early in pregnancy and might share similar mechanisms as those involved in defective placentation [5,6]. Assisted reproductive technologies (ART) have been associated with an increase in placental abnormalities [7–10]. In particular, the aberrant expression of genes involved in vessel development, trans-membrane transport, metabolism and oxidative stress has been found to occur in IVP placentae [9, 11– 13]. These biological processes are closely associated with critical placental functions, such as the transfer of nutrients and waste products between mother and fetus, suggesting that low nutrient condition is a hallmark of pregnancies obtained by ART. Although it is known that placental development is negatively affected by a lack of nutrients [14], detailed information about how placental cells respond to nutrient limitation is still lacking.

In various cellular settings, when nutrients are scarce, cellular metabolism shifts toward autophagy and recycling of cytosolic constituents [15,16]. This pro-survival mechanism could be involved in the response of trophoblast cells to stressors (i.e. starvation, hypoxia) affecting both normal and complicated pregnancies. In human placentae, expression of autophagic marker has been detected throughout the normal gestation, from the early stage until delivery [17–19]. High autophagic activity was also reported in placentae (collected at delivery) from pregnancies complicated by IUGR [20–22] and pre-eclampsia [23–25], suggesting an important role for autophagy in normal and abnormal placentation. Our previous studies demonstrated that *in vitro* culture of embryos is associated with vascularization defects in their placentae, and thus that ART affects embryo growth [26, 27]. Here we hypothesized that a high autophagic response in placentae from IVP embryos is induced to offset the reduced uptake of nutrients caused by the retard of placental vasculogenesis [27].

The aim of this work was to identify differences in cellular autophagy in early placentae derived from IVP embryos and those obtained after natural mating. First we looked for structural clues of autophagy, then we analysed a panel of genes and proteins regulating autophagy (*ATG5*, *ATG9*, *LC3*, *BCN1*, *RAB7*) whose expression in trophoblastic cells were previously reported [22], we analysed factors involved in the autophagic pathway (i.e. *BCL2* and *NIX*) as well in apoptosis and angiogenesis [28– 30], both required for proper placental development.

## Results

**Fig 1:**
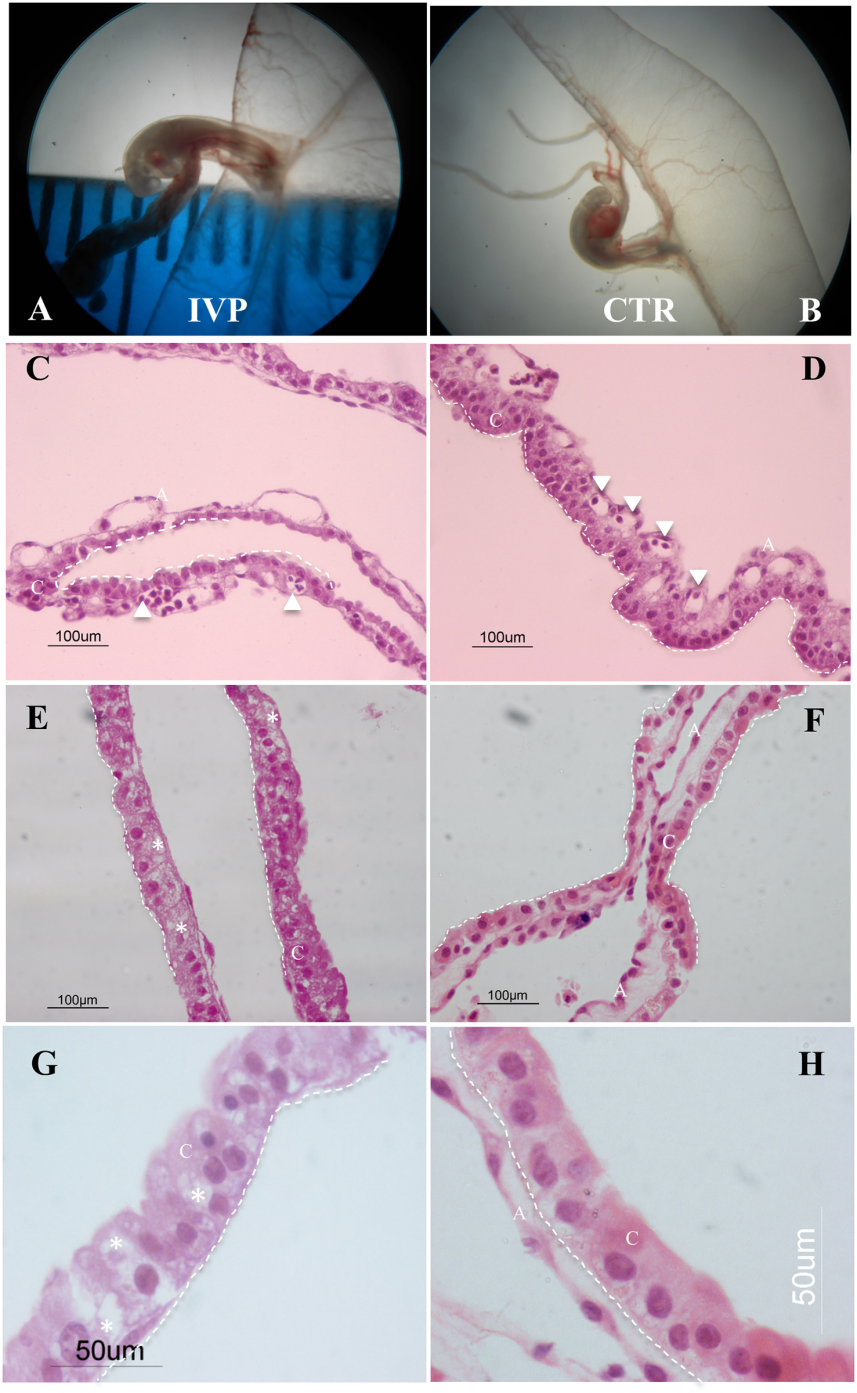
General view of IVP and CTR sheep embryos and their placentae (chorioallantoic tissue) at day 20 of sheep pregnancy. (A-B) IVP and CTR fetuses with their placentae under a stereomicroscope. (C-H) Hematoxylin & eosin staining of IVP and CTR placentae. Chorion (C) from IVP placentae (C-F; 40x) is less organized compared to that of CTR. In particular trophoblastic cell lose their epithelial-like structure, characterized by a columnar shape, and assume an irregular structure. (trophoectodermal epithelium denoted by dashed line; white arrowheads denotes vessels in the allantoic portion (A) of the placenta. At higher magnification (G-H; 100x) trophoblastic cells of IVP placentae show an amorphous shape without proper nucleus polarization and an highly vacuolated cytoplasm (vacuoles denoted by white star) compared to CTR.

**Fig 2:**
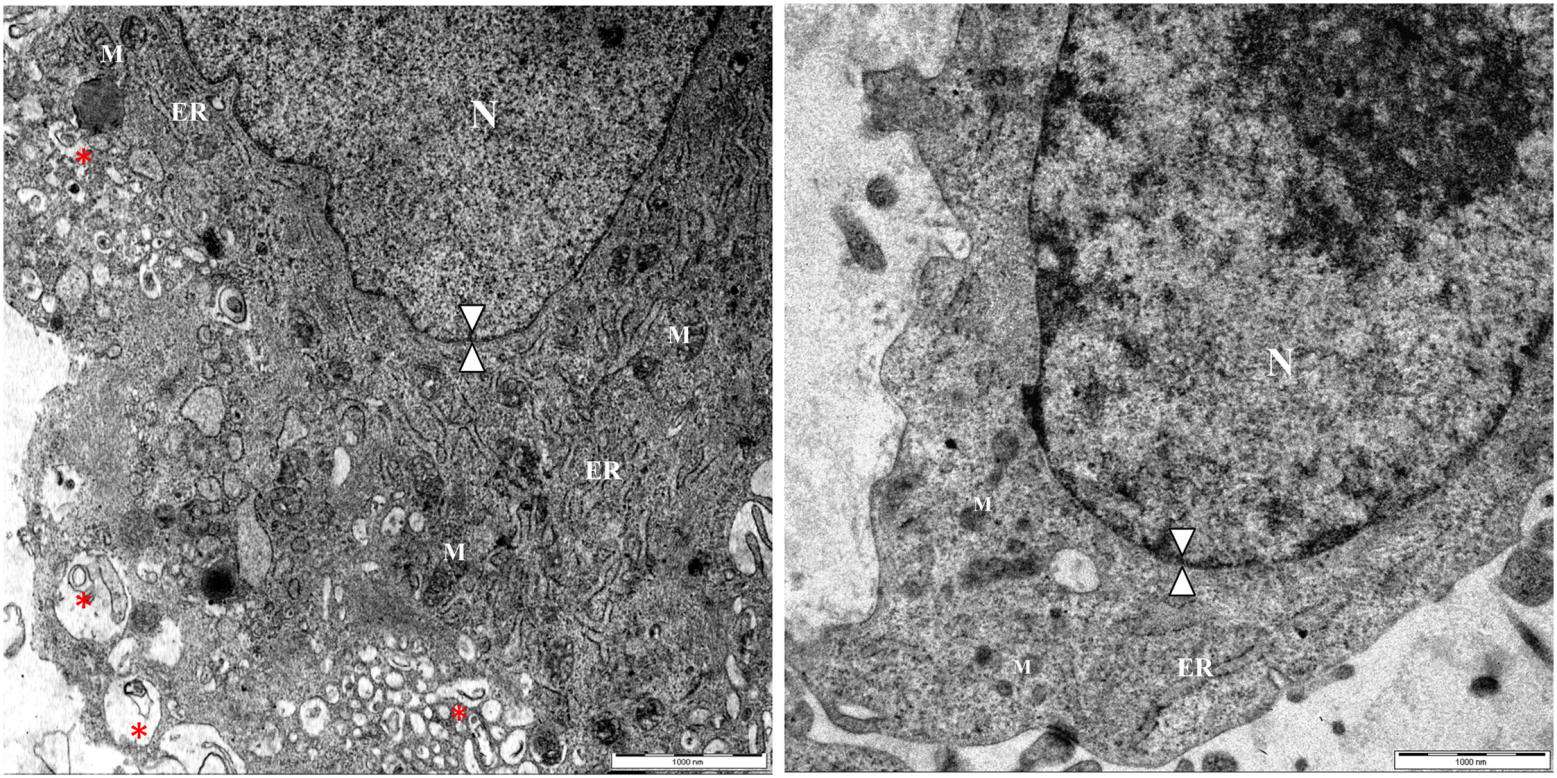
Structural evidence of autophagy in placentae from IVP pregnancies. Ultrastructural characterization of IVP (A) and CTR (B) placentae revealed that IVP placentae display the typical features of autophagy: a high degree of cytoplasmic vacuolization, filled autophagosomes (red star) and an intact nuclear membrane (double arrow head). N = cell nucleus; M = mitochondria; ER = endoplasmic reticulum.

Stereomicroscope observation of n = 8 IVP and n = 9 CTR conceptuses at day 20 of development revealed similar size and gross morphology (Fig 1A and 1B). Embryos of both groups were at a comparable developmental stage (Carnegie stage 14). Histological observation of the majority (>60%, 6/8) of IVP placentae showed severe anomalies in their characteristic chorio-allantoic structure. In particular, a substantial disorganization of the epithelial layer was found in the chorion and heavily vacuolated cells (Fig 2C and 2F). Instead of a columnar arrangement of trophoblastic cells forming organized layers as in CTR placentae (Fig 1D and 1F), the trophoblastic layer from IVP placentae was amorphous and disorganized (Fig 1C and 1E). In the details, trophoblastic cells lose the epithelial-like appearance and assume an irregular shape without proper nucleus polarization and intense cytoplasmic vacuolization (Fig 1G and 1H).

**Fig 3:**
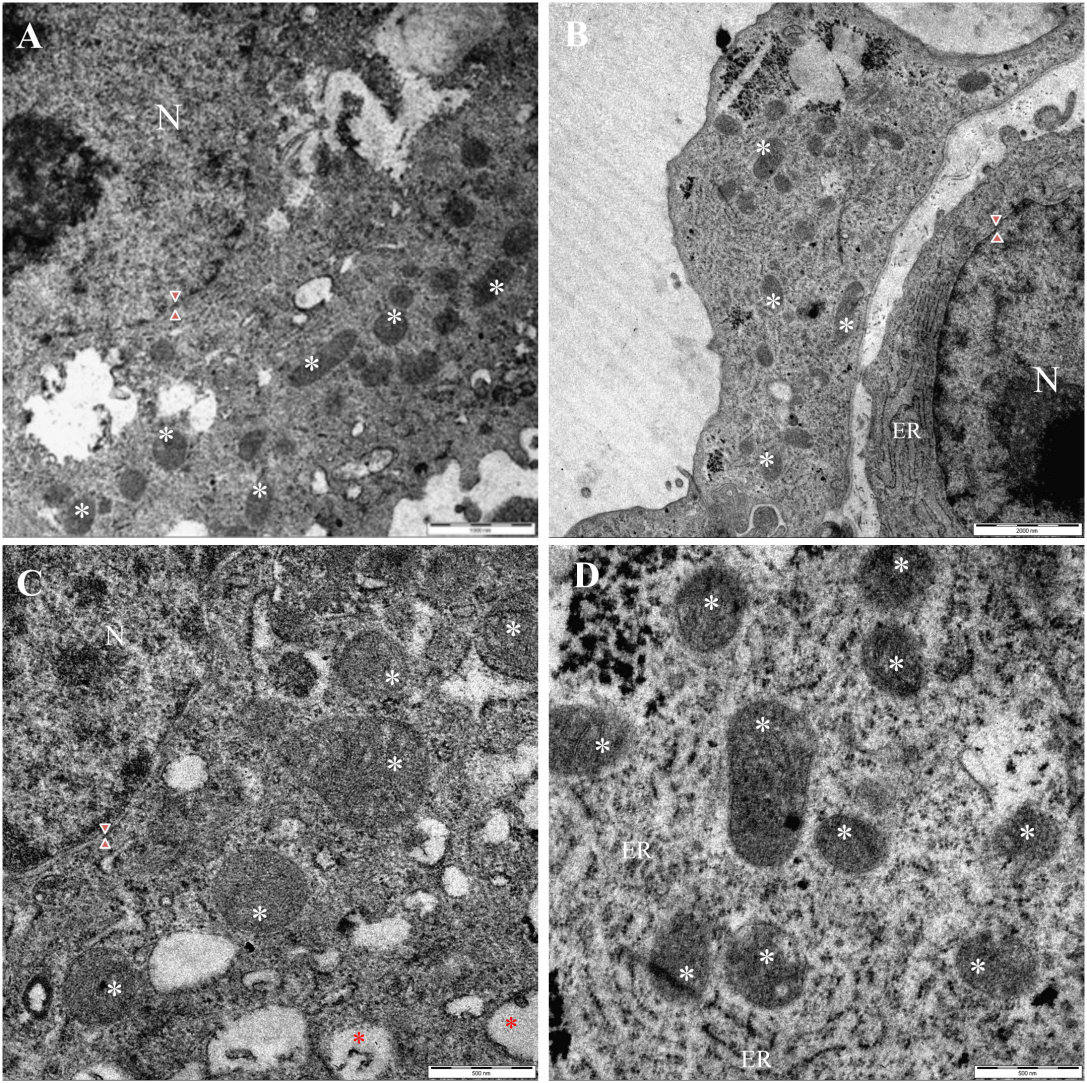
Mitochondrial structure and functionality in placentae from IVP pregnancies. Ultrastructural analysis displayed a comparable distribution of mitochondria (white star) in IVP (A) and CTR (B) placentae notwithstanding IVP mitochondria were swollen (C) than mitochondria in CTR cells (D). Red star denotes cytoplasmic autophagosome whereas red arrow heads indicates intact nuclear membrane.

**Fig 4:**
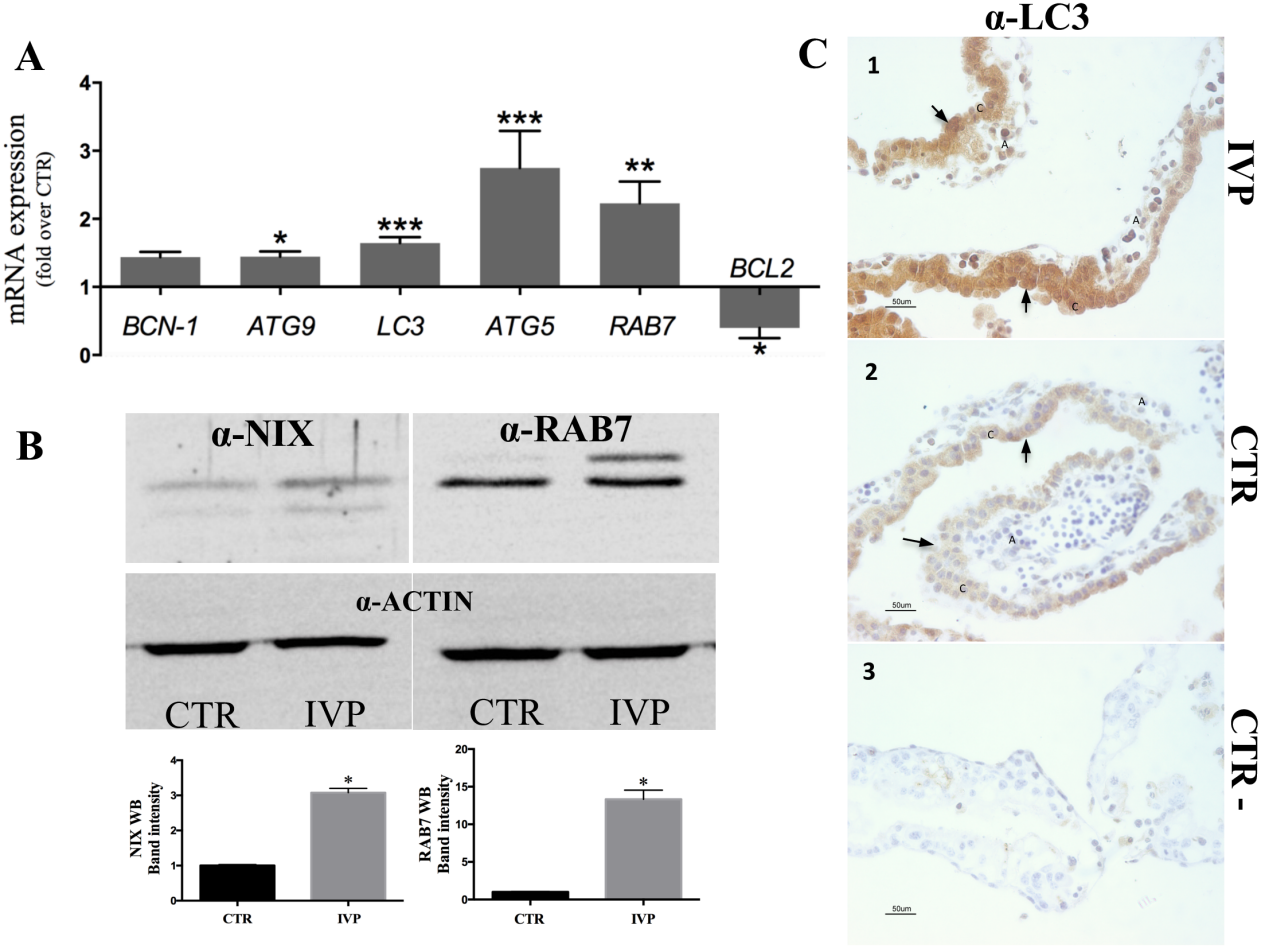
Molecular markers of autophagy in early IVP placentae. (A) Highly upregulated expression (*p<0.05; **p<0.005; ***p<0.0001) of genes regulating different steps of autophagy in IVP placentae. Note also low expression levels of the antiapoptotic gene, *BCL2*, which decreases autophagy through BCN-1 interaction. (B) Mitochondria extract from IVP placenta showed an elevated expression of the receptor protein for mitophagy recepror protein NIX. (B) Immunoblotting for canonical marker of autophagy, RAB7 (regulating autophagosome maturation) demonstrated elevated protein expression. (C). Immunostaining of LC3 (marker of autophagy induction) showed an intense and widespread LC3 expression in cells from IVP placentae (1), whereas in CTR placentae LC3 expression seems to be confined to the chorion (2). We used immunohistochemical assay because commercially available antibody against LC3 does not work in sheep under immunoblotting condition. In the figures black arrow shows positive staining of LC3, C denotes the chorion and A indicates the allantois. Control staining without primary antibody (3).

Ultrastructural investigation using transmission electron microscopy (TEM) revealed that the IVP placentae were characterized by a high percentage (35.08% (20/57), p <0.001) of autophagic cells. The most prominent morphological feature of autophagy in IVP placentae was the high number of double membrane-enclosed vesicles (autophagosomes) with engulfed portions of cytoplasm (CTR: 0.83 +/-0.54 vs IVP: 12.8 +/- 2.43; p = 0.0043) (Fig 3A). Autophagic cells from IVP placentae had intact nuclear membranes (Fig 3A) and enlarged endoplasmic reticula (Fig 3A). In addition, mitochondria in IVP placentae were swollen (Fig 4C and 4D), although present in similar quantities (CTR: 14,92 +/-1,361 vs IVP: 15,55 +/-2,834). Furthermore, there was an increased expression of the mitophagy marker NIX (Fig 4F) in mitochondrial protein extract from IVP placentae.

mRNA and protein expression of autophagy-related genes was generally upregulated in IVP placentae (Fig 4). Increased expression, with the only exception for *BCN-1*, was confirmed for genes (Fig 4C) and protein (Fig 4A and 4B, p = 0.019; Fig 4D) involved in all stages of autophagy: from initial step of vesicle elongation (*ATG9*: *p<0*.*05; LC3*, *ATG5*: p<0.001) to autophagolysosomal fusion (*RAB7;* p<0.005). Conversely, expression of antiapoptotic gene *BCL2*, involved in autophagy inhibition mechanism was less in IVP than CTR placenta (Fig 4C).

Next, we performed immunolabelling of the LC3 protein, which has been found highly expressed in both the chorion and allantois of IVP placentae, compared to CTR placenta where a mild LC3 expression seems to be confined in the chorionic part of the placenta (Fig 4D). Limited antibody available for our animal model, forced us to choose the technique with more reliable results, even though the immunoblotting is the recommended methods in these cases [31].

## Discussion

Our results have shown an increase in autophagy activation in placentae after transfer of IVP embryos on Day 20 of pregnancy in sheep. In the light of our previous results demonstrating impaired vasculogenesis in IVP placentae [27], high placental autophagy seems to be a compensatory mechanism to support foetal growth in a low nutrient condition.

Impaired glucose and oxygen levels in human trophoblastic cells *in vitro* cause dysfunction of mitochondria and the endoplasmic reticulum and lead to autophagic pathway activation [17]. Auto-digestion of intracellular organelles can temporarily sustain energy production and thus prevent cell death. At the same time, excessive autophagy can lead to the destruction of essential proteins and organelles beyond a certain threshold, thus resulting in cell death [32]. Mitochondrial degradation shifts the cellular fate toward death [33]. Here, autophagy seems to play an adaptive role in IVP placentae at day 20 of pregnancy, fostering cellular survival rather than death, as demonstrated by the comparable number of mitochondria found in IVP and CTR placentae. On the other hand, mitochondria from IVP placentae appeared more swollen than mitochondria from CTR. Mitochondrial protein NIX, involved in the recently discovered pathway of mitophagy [34], was also highly expressed in IVP placentae in our study. NIX triggers mitochondrial depolarization, which in turn causes large amplitude swelling and release of proapoptotic proteins into the cytosol. With moderate stressors, mitophagy can act as a repairing mechanism to eliminate damaged mitochondria. When stress increases, mitophagy may no longer contain proapoptotic factors being released from mitochondria [35]. For this reason NIX is considered as autophagy regulatory gene with a crucial role in cell fate. Interestingly Mizushima [36] and others [30] demonstrated that in a nutrient rich condition LC3 overexpression is not sufficient for autophagy induction in both *in vitro* (cancer cell) [36] and *in vivo* (skeletal muscle) [30] models. On the contrary, in both normal and starved condition autophagosome formation in skeletal muscle is in vivo induced by NIX overexpression as well as the induction of autophagy is significantly decreased by NIX knockdown [30]. Similar results were obtained in cardiomyocytes in a cell culture model of ischemia-reperfusion injury [37] and in normal and cancer cells cultured in normoxia and under hypoxic condition [29].

Therefore NIX overexpression per se seems to be sufficient to trigger autophagy while transcriptional regulation of other autophagy-related-genes is necessary for the maintenance of the already activated autophagy.

Morphological observation of autophagic clues, such as cytoplasmic vacuolization, swollen endoplasmic reticula and mitochondria, as well as increased levels of NIX are indicative of deregulated autophagy in IVP placentae. However, it may not answer the question of whether autophagy is *blocked* at a particular stage or *hyperactivated* in early IVP placentae. Both the hyperactivation and the blockage of autophagy at any particular stage would similarly result in the accumulation of vacuoles and defective organelles. In this case, given the technical limit of the experimental model, the answer may be provided by gene expression analysis.

In mammalian cells, upregulated expression of *ATG5* indicates enhanced autophagy activity, as demonstrated in mice overexpressing *ATG5* [38], and elevated level of RAB7 is required to complete autophagosome maturation (i.e. during last step of autophagy) in ovarian cultured cells [39]. Furthermore, general overexpression of a great majority of canonical autophagy markers, that regulate the process from the initial steps of autophagosome formation [40](i.e. LC3 mRNA and protein) through final step of autophagosomes-lysosomes fusion [39] (i.e. RAB7 mRNA and protein), suggests a hyperactivation rather than a blockage of the autophagic process.

From all selected autophagy genes, there was only one gene (*BCN1*) whose expression was not deregulated. In fact, although *BCN1* is known to induce autophagy, it is debated whether its expression should change in response to an autophagic stimulus. For example, different human studies focused on preeclamptic pregnancies showed divergent results for BCN1 expression: In one case BCN1 was found to be increased in placental tissue from preeclamptic pregnancies at delivery [41] whereas Oh and collegues reported no change in BCN1 expression in term placentas from patients with preeclampsia compared with normal ones [25].

Otherwise, a positive feedback for autophagy activation could be triggered by downregulation of the antiapoptotic gene *BCL2*, known to decrease autophagy through BCN-1 interaction in yeast such as in mammalian cultured cells and tissues [28]. Interestingly, the BCL-2/BCN1 complex represents a convergence point between apoptosis and autophagy [28], depending on the nutrient status of the cell [42]. As demonstrated in multiple cell lines [28, 43] and in mice [42], down-regulation of BCL-2 enhances autophagy activation through releasing BCN1 from the BCN1/BCL2 complex, which inhibits autophagosome formation [42, 43].

Moreover, competitive binding of BCL-2 to other BH3-only proteins, also prevents BCN1 inhibition and induces autophagy [44, 29]. In particular, in normal and cancer cell, BCN1/BCL2 complexes can be disrupted by NIX/BNIP3L, which bind to BCL-2 proteins and subsequently activate the autophagy initiation complex [29]. During cellular stress condition, such as hypoxia, NIX overexpression is involved on the autophagic uptake of mitochondria [45]. In moderate stress condition NIX represent a survival mechanism but excessive insults leads to mitochondrial damage and consequently to cell death [46]. Therefore gene expression data from IVP placentae, showing *BCL2* down regulation associated with NIX overexpression, support our hypothesis that despite its temporal limitations, placental autophagy contributes to the compensatory growth of the fetus developed from IVP embryo.

Placental autophagy as an adaptive strategy has been implicated as a means of safeguarding the normal brain development of mice fetuses in a model of short-term food deprivation [47]. In that model, nutrient depletion significantly decreased the expression of maternally-imprinted *PEG3* in the placenta, while increasing its expression in the hypothalamus. This inverse relationship of *PEG3* expression is linked to autophagy and ribosomal turnover in the placenta, which in turn sustain the nutrient supply for the developing brain.

There is a growing body of literature suggesting that increased cellular autophagy has been associated with the inhibition of angiogenesis, [48–50]. In vitro studies demonstrated a *PEG3*-dependent autophagy in vascular endothelial cells [49]. Migration of these cells is responsible for neovascularization and vessel morphogenesis, however it has been demonstrated that the induction of *BCN1* and formation of PEG3-BECN1 complexes might be critical for blocking both migration and morphogenesis [49]. Decorin (DNC), a component of extracellular matrix, inhibits angiogenesis in vitro by antagonizing vascular endothelial growth factor (VEGF). DCN binds to VEGFR2 and activates a signal cascade that involves the transcriptional regulation of *LC3* and *BCN1* [48, 50]. DCN binding to VEGFR-2 was demonstrated also in human ExtraVillous Throphoblast cells [48, 51] with strong implications for placental physiology and pathology because of its dual role on the trophoblast and endothelial cells. Therefore, our previous data about retarded vasculogenesis and low VEGF expression in IVP placentae at day 20 of sheep pregnancy [27] can be interpreted as an upstream event to evoke autophagy.

Previously, human studies on pregnancies complicated by preeclampsia have shown that impaired autophagy of extravillous trophoblasts may induce poor vascular remodelling [52,53]. More recent, Nakabayashi and other demonstrated that impaired autophagy in EVT were evident in oocyte donation pregnancies obtained by in vitro fertilization, regardless of the presence or absence of preeclampsia [54]. However, in both case, data were obtained from third trimester placenta while we focused on early development when a correct feto-maternal interaction is necessary for normal pregnancy proceeding. To our knowledge, there is only one study that reported an increased autophagy during early pregnancy in villous sample collected from spontaneous miscarriage [19]. However is not determined if autophagy has been induced as consequence of fetal demise, whereas in our study all analysed samples were collected from alive foetuses.

In conclusion, our data demonstrated increased autophagy in early pregnancy obtained following transfer of IVP embryos. This strongly suggest that the placenta provides the first line of defense against fetal starvation. Placental autophagy may act as a survival mechanism to ensure further development of growth restricted fetus by transforming the sequestered material into amino acids and other simple molecules, which are then used as nutrient sources. However, this mechanism is limited to short-term and minor deficiencies and cannot replace a severe and/or long-lasting lack of nutrients.

## Materials and Methods

All chemicals, unless otherwise indicated, were obtained from Sigma Chemical Co. (St. Louis, MO, USA).

### *In vitro* embryo production

All animal experiments were performed in accordance with DPR 27/1/1992 (Animal Protection Regulations of Italy) in concordance with European Community regulation 86/609 and were approved by CEISA (Inter-Institutional Ethics Committee for Animal Experimentation) Prot. 79/2013/CEISA Prog. 58. Protocols for *in vitro* maturation, *in vitro* fertilization and embryo culture were adapted from those previously described from our laboratory [26, 27]. Briefly, sheep ovaries (from fertile Sardinian ewes, age: 4–7 years) were collected from local slaughterhouses and transferred at 37°C to the laboratory within 1–2 h. Oocytes were aspirated from ovaries, selected and then *in vitro* maturated in bicarbonate buffered TCM-199 (Gibco, Life Technologies) containing 2 mM glutamine, 0.3 mM sodium pyruvate, 100 μM cysteamine, 10% fetal bovine serum (FBS) (Gibco, Life Technologies), 5 μg/ml ovine FSH (Ovagen, ICP, Auckland, New Zealand), 5 μg/ml ovine LH and 1 μg/ml estradiol. Maturation was conducted in a humidified atmosphere of 5% CO2 in air at 39°C for 24 h. Matured oocytes were partially denuded and *in vitro* fertilized with semen from Sarda breed rams (Semen-Italy, Italy). Presumptive zygotes were transferred into 20 μl drops of synthetic oviductal fluid (prepared in our laboratory) enriched with 1% (v:v) minimum essential medium (MEM) nonessential amino acids (Gibco, Life Technologies), 2% (v:v) basal medium Eagle (BME) essential amino acids, 1 mM glutamine, and 8 mg/ml BSA covered with mineral oil. Culturing was carried out in a humidified atmosphere of 5% CO2, 7% O2 and 88% N2 at 39°C. On day 3 and day 5 of culture (where day 0 = day of fertilization), the medium was changed.

### Embryo transfer and sample recovery

Sardinian sheep were synchronized with 25 mg Chronogest sponges (Intervet, Milan, Italy). Sheep were divided into two groups: the first were recipients of *in vitro*-produced embryos (IVP), and the second were mated naturally (control—CTR), n = 10 and n = 7, respectively. At day 6 of culture, expanded blastocysts (2–4 per ewe, depending on embryo quality determined following IETS manual [55]) were transferred surgically by paramedian laparatomy to recipient sheep in the IVP group 6 days after estrus. For both the IVP (8 collected from 7 ewes; pregnancy rate 70%) and CTR (9 collected from 6 ewes; pregnancy rate 85%) groups, fetuses and placentae were surgically recovered from sheep under general anesthesia, on day 20, as we described previously [26, 27]. Once collected in Petri dishes (90 mm) foetuses were observed under the stereomicroscope to assess their vitality by their heartbeat. Chorion-allantoids (Fig 4) were snap-frozen in liquid nitrogen for subsequent gene and protein expression analysis, fixed in 4% paraformaldehyde in PBS for histological analysis or fixed in 2.5% glutaraldehyde for ultrastructural analysis.

**Fig 5:**
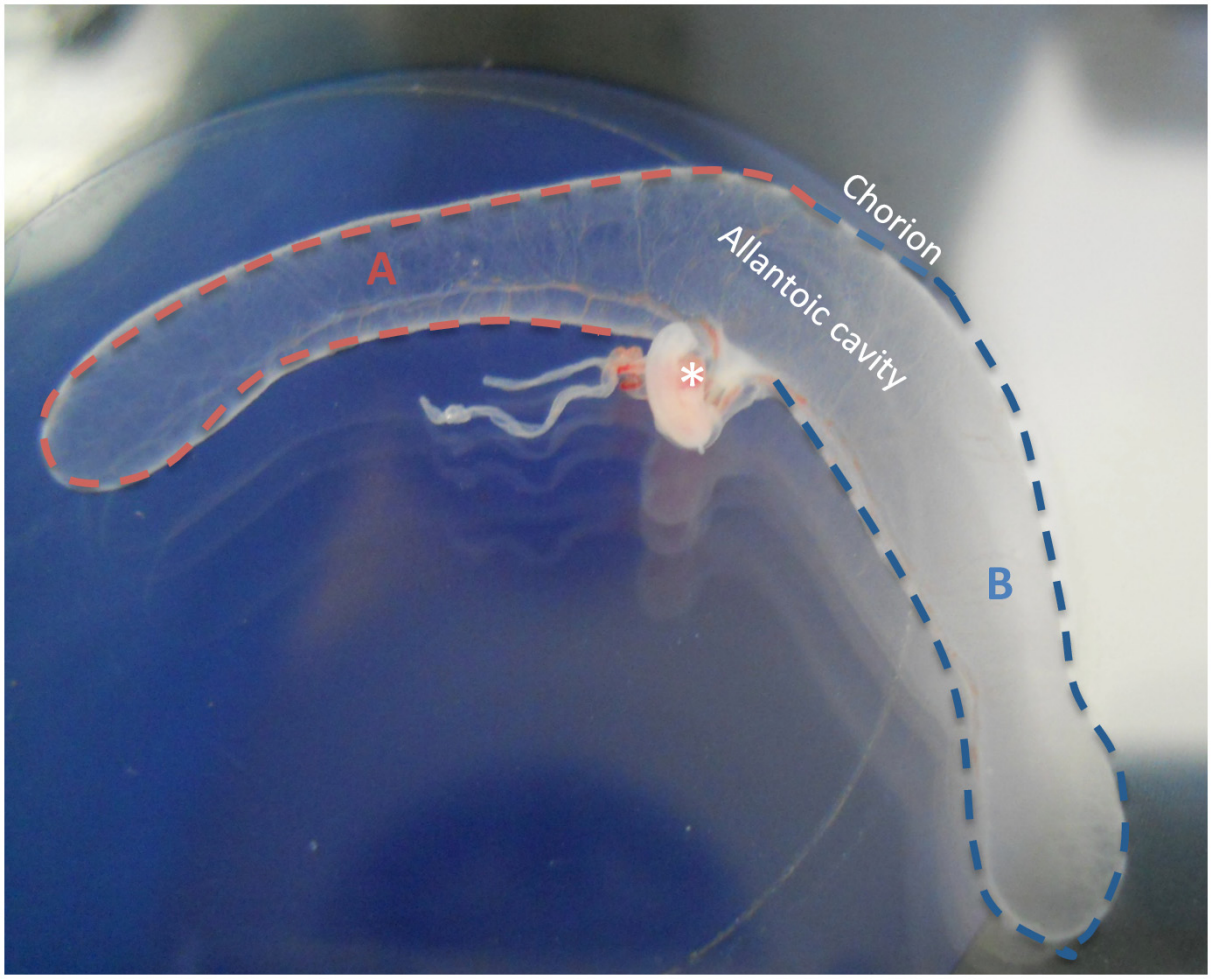
Macroscopic view of sheep embryo with placenta collected on day 20 of pregnancy. The asterisk indicates the embryo inside the amniotic sac. The placenta (chorioallantoic tissue) was cut into two similar parts, indicated as A and B. One part was fixed for histological, IHC and TEM evaluation and the other was snap frozen for gene expression analysis (qPCR and Western blotting).

In our experimental settings, due to the limited size of such tissues at this developmental time point, all collected placentae (both IVP and control) were divided into two parts (Fig 5): one part was subjected to molecular analysis and the other, to structural (ultrastructural and histological/immunological) testing of cellular autophagy.

### Histological analysis

Placental tissue (n = 8 for IVP; n = 9 for CTR) collected on day 20 of gestation were fixed in 4% paraformaldehyde overnight, dehydrated with increasing ethanol grandient (5’ each step), cleared in xylene mixture (5’ minute each step) and finally paraplast embedded. 5 μm sections were dewaxed and rehydrated. Then, one part was used for hematoxylin eosin staining and other for immunohistochemistry analysis. First group of the sections were stained with Hematoxylin Solution (Bio-Optica, Milan, Italy), differentiated in acid alcohol solution, counterstained with Eosin Solution (Bio-Optica, Milan, Italy) and then dehydrated in 100% ethanol and cleared in xylene mixture. For immunohistochemistry, tissue sections were incubated overnight at room temperature with 4 μg/ml of primary rabbit polyclonal antibody against LC3 (ab58610; Abcam, Cambridge, UK). Negative control:tissue sections were overnight incubated without primary antibody. Antibody binding was visualized using Universal LSAB^™^ Kit/HRP and Dako Liquid DAB Substrate Chromogen (Dako, Glostrup, Denmark). Images were captured using a Nikon Eclipse E600 light microscope (Melville, NY, USA).

### Transmission electron microscope

Placental tissues (n = 5 for IVP; n = 5 for CTR) were fixed in 2.5% glutaraldehyde for 24 h and post-fixed in 2% OsO4 for 4 h. Samples were dehydrated through a graded series of EtOH and infiltrated with Epon resin in 100% acetone, infused twice for 1 h in pure epon resin and polymerized for 24 h at 65°C. A LEO 912AB electron microscope (Leo Electron, Thornwood, NY, USA) was used to examine 60 nm sections. Images were captured by the Slow Scan CCD (Proscane) using EsiVision Pro 3.2 software (Soft Imaging Systems GmbH).

Morphological changes induced by autophagy were evaluated in TEM images. Micrographs of single cells were taken by random screening (at least 50 cells/group). Cells with two or more visible autophagosomes were considered to be autophagic. The number of these cells was divided by the total number of cells in the same field to generate the percentage of cells undergoing autophagy.

The number of autophagosomes/mitochondria per cell was plotted against the percentage of the cell population containing this number of autophagosomes. Data represent the average of all fields.

### Western immunoblotting

Total protein and mitochondrial protein extracts were obtained from IVP and CTR placental tissues (n = 3 each group) using RIPA buffer and Mitochondrial Isolation Kit for Tissue (Abcam) respectively. Then proteins (30μg of total extract for Rab7 and 10μg of mitochondrial extract for Bnip3L/NIX) were denatured by heating at 95°C for 5 min in 1% (v:v) sodium dodecyl sulphate (SDS), 1% (v:v) β-mercaptoethanol, 20% (v:v) glycerol in 50 mM Tris–HCl at pH 6.8. Samples were subjected to electrophoresis in 10% SDS polyacrylamide gels. After electrophoresis proteins were transferred to nitrocellulose membranes. Membranes were blocked in TBS-T (0.2% (v:v) Tween-20 in 20 mM Tris, 137 mM NaCl at pH 7.6) with 5% (w:v) skimmed milk, and then washed three times in TBS-T at room temperature. Membranes were incubated with the primary rabbit polyclonal antibodies anti-RAB7 (1:300; ab77993, Abcam), anti-Bnip3L/NIX (1:300; ab8399, Abcam); anti-Actin antibody was used as loading control (1:1000; sc-1615, Santa Cruz Biotechnology, Santa Cruz, USA) diluted in 0.1% blocking solution at 4°C overnight (O/N). After three washes with TBS-T, membranes were incubated at room temperature with the secondary antibodies (1:1000) in 0.1% blocking solution for 1 h. After three washes in TBS-T, the final detection was performed by enhanced chemiluminescence using the ECL Plus Western Blotting Detection System (Promega, Milan, Italy). Image acquisition was carried out using the ChemiDoc System (Bio-Rad, Milan, Italy). Using ImageJ densitometry software, differential gel band intensities of CTR and IVP samples were quantified to derive the relative -fold increase or decrease. Semi-Quantitative densitometric analysis was performed on the specific bands (The approximate 40kDa and 23KDa top bands represents monomer of Bnip3L and Rab7 respectively) based on their relative intensities.

### mRNA expression analysis

Total RNA from IVP and CTR placentae (n = 6 in both group) was extracted using an RNeasy Mini Kit (Qiagen, Milan, Italy) according to the manufacturer’s instructions. Total RNA integrity was assessed by a 2100 Bioanalyzer (Agilent Technologies, Waldbronn, Germany). Samples with an RNA Integrity Number of at least 8.5 were reverse-transcribed using GoScript^™^ Reverse Transcription System (Promega, Milan, Italy) and used for gene expression analysis using specific 5’-3’ primer pairs designed to anneal at 56/58°C with amplification efficiency (E) range between 2.1 and 1.9 (available on request). Real time PCR have been carried out using Sso Advanced Universal SYBR green Supermix (Bio-Rad, Milan, Italy) with CFX Connect Real-time PCR detection system (Bio-Rad, Milan, Italy), according to the manufacturer’s instructions. Relative gene expression data were calculated using the comparative threshold cycle method (ΔΔCt) with μ*TUBULIN* and *SDHA* as housekeeping genes. To check the specificity of each amplification, dissociation analysis have been performed into every run.

### Statistical analysis

Statistical analysis was performed using Instat 5 (GraphPAD software for science, San Diego, CA, USA). Data reported are the mean±S.E.M. and were analyzed using the non-parametric Mann-Whitney test (Fig 4A, 4B and 4D). Data expressed as percentages were analyzed using the Fisher’s exact test. Only p values <0.05 were considered significant.
